# Beyond the border of the athlete-centered approach: a model to understand runners' performance

**DOI:** 10.3389/fpsyg.2023.1137023

**Published:** 2023-08-24

**Authors:** Mabliny Thuany, Thayse Natacha Gomes, Katja Weiss, Beat Knechtle, Ramiro Rolim, Marcos André Moura dos Santos

**Affiliations:** ^1^Centre of Research, Education, Innovation and Intervention in Sport (CIFI2D), Faculty of Sport, University of Porto, Porto, Portugal; ^2^Post-Graduation Program of Physical Education, Department of Physical Education, Federal University of Sergipe, São Cristóvão, Sergipe, Brazil; ^3^Department of Physical Education and Sport Sciences, University of Limerick, Limerick, Ireland; ^4^Department of Physical Education and Sport Sciences, Physical Activity for Health Cluster, Health Research Institute, University of Limerick, Limerick, Ireland; ^5^Institute of Primary Care, University of Zurich, Zurich, Switzerland; ^6^Medbase St. Gallen Am Vadianplatz, St. Gallen, Switzerland; ^7^Associated Postgraduate Program in Physical Education, University of Pernambuco and Federal University of Paraiba, Recife, Pernambuco, Brazil

**Keywords:** ecological systems, cross-cultural research, endurance running, athletes' performance, coach

## Abstract

Our purpose is to move beyond the borders of the athlete-centered approach by examining the runners' environment interplay as a key factor for performance. Based on the ecological systems theory, the micro-level (intrapersonal, interpersonal, and training characteristics), meso-level (a direct association with athletes is not observed, but the environment plays an influence on the relationships built at the micro-level), and macro-level (contextual features that influence athletic systems) were theorized and contextualized as important factors for the expression of different outcomes, including performance and participation. We also conceptualized the microtime, mesotime, and macrotime as a constraint. Through this model, we aimed to provide applications and conclusions about how this conceptual model provides advances in the scientific research field. By understanding how environmental factors influence their performance, runners can make informed decisions about where and how to train and compete. Furthermore, by recognizing the role of culture and social context in shaping runners' experiences and outcomes, we can work toward creating a more equitable and supportive running culture for all.

## 1. Introduction

“A wonderful harmony arises from joining together, the seemingly unconnected” (attributed to Heraclitus of Ephesus c. 500 BC).

Sport is a global phenomenon and has been integrating the contemporary debate about sustainability, peace, and human development (Lemke, 2016). The relevance of sports for nations is highlighted through two main perspectives: social and economic. As a social phenomenon, sport has the potential to help in the reduction of inequalities, empowering minorities, increasing national pride through megaevents, and contributing to social cohesion within and between communities (Spaaij, 2009). Sport is also a global market, potentially contributing to the countries' development, for example through the synergies between tourism, industry, and transport sectors (Rázvan et al., 2020).

As an expression of human excellence, sports performance is also debated in the scientific literature. The interest to improve athletes' performance, through the comprehensiveness of factors that explain/predict performance, has increased among sports science researchers (Yan et al., 2016). As a dynamic, non-linear, and multidimensional phenotype, there seems to exist a consensus that athletes' performance should be investigated through a holistic approach (Balague et al., 2013; Balagué et al., 2017). However, the athlete-centered approach (Midgley et al., 2007) overvalues individual characteristics, such as physiology, psychology, genetics, and biomechanics, as the key factors to understand athletes' performance (Moir et al., 2019; Zani et al., 2022)—dominating the scientific debate for a long time.

The athlete-centered approach is intrinsically related to the mechanical idea that compares the human body with a machine, where each part must be understood separately to provide the answer about the “final product” (Capra, 2012). Notwithstanding the relevance of this approach to comprehensiveness regarding the body mechanisms and functions (Kent and Hayes, 2021), sports performance cannot be fully understood if the subject–environment relationship is not considered. Furthermore, since the subject–environment relationship operates in an open system, the use of holistic approaches to understand the behavior that emerges from this interaction is necessary.

Sports performance is also context- and sport-dependent. These fundamental premises postulate that sports performance (at the individual or country level) reflects the own resources available at a specific timeframe (i.e., intrapersonal, interpersonal, infrastructure, and policy initiatives) to correspond to the requirements to be successful in a specific sport (Knuepling and Broekel, 2022). Furthermore, since the athletes' opportunities to be engaged in specific sports practices reflect the inputs (i.e., financial and moral support, sports practice opportunities, sports culture, and geographic features) available in athletes' day-life (Santos et al., 2019), the role of the environmental factors that can be transferred and expressed in terms of performance should be considered.

As part of the sports science field of study, the evolution of endurance sports in a scientific context was strongly influenced by the athlete-centered approach. Although the advances from these researchers, we believe that the field of study could be benefited from an approximation with an ecological approach, considering the proximal to the distal factors that can be contextualized to deeply understand the performance at the individual or country level. For example, much has been discussed about the factors that explain the success of East African runners; however, few studies considered a more contextual approach to deeply understand this phenomenon. For that, we theorized and contextualized running performance, considering both individual and environmental factors through a holistic approach.

## 2. Moving beyond the athletes- centered approach

The nature–nurture dichotomy is a long-standing debate in different fields of study, such as education, psychology, science, and sports (Klissouras, 2001; Knechtle, 2012; Yan et al., 2016). The belief that genetic characteristics are the most relevant factors to the expression of behavior was scientifically proposed by Galton (1875) when he postulated the impossibility of transposing genetics by training (Galton, 1875). On the other hand, behavioral theories highlight the role of the environment as the main actor in the expression of human development or the acquisition of a given skill (Simon and Chase, 1973; Ericsson et al., 1993).

In addition to the fact that this secular debate no longer finds support, since it represents a limited and deterministic approach to reality, and this dichotomization is still presented in some theoretical frameworks used in sports science research, through the use of analytical and monodisciplinary approaches (Loland, 2013; Balagué et al., 2017). For instance, since the 1960s, the comprehension of the African runner's phenomenon in the international context of running was deeply influenced by the analytic reductionism that highlights genetic, morphological, and physiological characteristics as the most important domains for performance prediction/expression (Kruger et al., 2012; Wilber and Pitsiladis, 2012; Moir et al., 2019). Narratives about genetic characteristics, morphology, and physiological parameters (i.e., maximum oxygen consumption, metabolic efficiency, and hematological parameters) were debated for many years (Wilber and Pitsiladis, 2012; Tucker et al., 2013). However, a recent comparative study investigating the relationships between ethnicities (Kalenji's—Kenya and Oromo—Ethiopia) and endurance running success concluded that causality inferences regarding the relationship between genetics and sports must be avoided, once it is both scientifically incorrect and prone to reinforcing population (racial) stereotyping (Hamilton, 2000; Zani et al., 2022). With this conclusion, the authors do not exclude the role of genetics but reinforce the importance to move forward.

The geospatial variation associated with the odds to be an elite athlete in specific places has been known as the “birthplace effect” (Smith and Weir, 2020; Faria et al., 2021; Leite et al., 2021; van Nieuwstadt et al., 2021). This phenomenon has been largely discussed and investigated in different contexts and/or sports modalities, such as sprinters (Jamaica), soccer players (Brazil), ice hockey (Canada), and endurance running (East Africa) athletes. From the set of factors usually highlighted as related to this phenomenon, the proximal features are highlighted, such as sports facilities, athlete–coach relationship, neighborhood security perception, opportunities to deliberate play, and pro-community behavior (Cote et al., 2006; Oishi et al., 2007; Balish and Côté, 2014; Wattie et al., 2018; van Nieuwstadt et al., 2021). However, few were discussed and contextualized considering the distal environment to the explanation of this phenomenon.

In the context of endurance running, aside from genetic, morphological, physiological, and training characteristics that contribute to the African runner's phenomenon (i.e., Kenya, Ethiopia) (Larsen, 2003; Zani et al., 2022), the country's historical background, the population's lifestyle characteristics, the economic development, and perspectives of social ascension and/or better living conditions through the sport need to be considered as factors related to the running training engagement and performance (Bale and Sang, 1996). For example, colonization, religious missions, and school policies were important agents in the dissemination of running in the Kenyan context. During the 1950s, British athletics coaches, athletes, and physical education teachers were designated to move to Kenya as part of an assistant program, working as role models for Kenyan runners, which was also called “cultural imperialism” (Said, 1997).

These factors highlight that Kenya, as an ‘international power' in long-distance running, was developed in the long term through a connection of different factors (Said, 1997) that do not always seem easily connected. As pointed out by Sniderman (2010), the dominance or absence of some populations/nations in some sports must be explained by the shared attitudes of most members of that population, as well as governmental and public initiatives that put the country in an international showcase. Once again, in the case of Kenyan runners, changes in immigration runners' rules and travel restrictions were of relevance to increase their participation in international events (Said, 1997). However, the magnitude in which changes at the distal level can be converted into better opportunities and conditions at the proximal level is not clearly mentioned in the scientific literature.

## 3. A theoretical framework for runners' performance understanding

### 3.1. The ecological systems theory

Bronfenbrenner's (1977, 2011) ecological systems theory was first presented in 1977. The theory was developed in the context of psychology as a critique of the experimental research and interventions in this field, aiming to provide a new approach to understanding human development. According to Bronfenbrenner, the best strategy to understand human development is a holistic approach involving the subject and the environment. The ecological systems theory allows us to understand the demands of human development beyond the direct observation of behaviors through an interaction between two people. In addition, it requires examining multi-person interaction systems, considering the environmental aspects beyond the immediate situation containing the subject (Bronfenbrenner, 1977). In this case, the ecological systems theory considers different levels, including the micro-level, meso-level, exo-level, macro-level, and chronosystem (Bronfenbrenner, 1977).

Even though the theory was not developed for sports scientists, it has been applied in different contexts of sports science. Studies about the parental role in youth sports involvement (Holt et al., 2008), psychosocial stressors in women athletes (Pascoe et al., 2022), sport development programs (Burnett, 2015), effects of club characteristics on basketball players' performance (Junior et al., 2019), and physical activity promotion (Spence and Lee, 2003) used ecological systems as a framework to answer their questions and guide the hypothesis. For athletes' process development, the commitment of stakeholders, clubs, place of residence, family, and support from friends was previously associated with performance (Henriksen et al., 2010; Smith and Weir, 2020). For example, Durand-Bush and Salmela (2002) indicated that the context in which athletes were inserted during general training significantly influenced sporting success. Moreover, access to facilities and equipment and support from friends, family, coaches, and staff were relevant for practice maintenance. For track and field, Henriksen et al. (2010) showed that sports clubs play an important role in athletes' development during the training period, highlighting that a strong organizational culture, characterized by values and integral development of the athlete, was crucial for athletes' development.

As the studies are mainly centered at the proximal level, with the purpose to understand skill acquisition and establishing the relationship with the subject–task–environment (Uehara et al., 2016; Glazier, 2017), previous studies highlighted the relevance to further explore the role of distal constraints in the expression of athlete's performance (Uehara et al., 2016). In the context of sports performance, differences in cultural and socioeconomic characteristics between countries can be expressed in terms of sports programs, national events, training facilities, and scientific research, providing different environments and conditions for the athlete's development. Countries are more likely to invest in sports in which they are well-represented at an international level, but as mentioned above the context of Kenyan runners, the sports context at the country level takes time and is still related to the historical background.

Thus, it is important to understand country-specific environments, which act differently on runners' development, as well as the best model to build a friendly environment to promote athletes' performance, including cultural investigation. Since sports science, as a study field, was strongly influenced by a positivist paradigm, research questions and methods were also strongly related to a quantitative approach, interested in unidirectional causality between independent and dependent variables. These characteristics have led to the use of individual approaches to answer most of the research questions in this study area. However, when the interest is centered in to change the level of analysis from the individual level to the group level, cultural factors should be considered. Since cultural factors are important drivers in a country's engineering, the shared beliefs, traditions, costumes, and values of the group/community (Shiraev and Levy, 2010) can be linked to the emergent patterns that differentiate one successful athlete from a successful group of athletes.

### 3.2. Cross-cultural psychology

Cross-cultural psychology is the scientific study of the variation in human behavior considering differences under cultural conditions and also understanding how cultural practices evolve and affect human behavior in a bidirectional relationship (Shiraev and Levy, 2010). However, cross-cultural research is not only concerned with differences between countries but also with similarities (Shiraev and Levy, 2010). Cross-cultural psychology is related to several population-level disciplines that are not only concerned with individual approaches but also incorporate different domains that comprise social behaviors, personality, and group perception. Previous research on this topic examined behaviors across cultures, as shown by Inman et al. (2017), where cultural values were related to alcohol consumption, and by Cheng et al. (2013), whose results showed that external locus of control and anxiety symptoms were weaker for collectivist societies compared to individualist societies.

Cross-cultural psychology is operationalized through cross-cultural research. Cross-cultural research is used to compare the studies of cultures or countries (Buil et al., 2012). In general, the number of studies using cross-cultural approaches has increased in the last few years. This increase is related to technological advancements, migratory streams, and globalization (van de Vijver and Leung, 2021). Specifically in the sports context, this topic is embryonic, with most of the studies focusing to perform cross-cultural validation of instruments (Arthur et al., 2022; Dos Reis-Junior et al., 2022). In addition, original studies have barely explored the potential of culture to explain different behaviors/outcomes in different contexts (Balish et al., 2016). A cross-cultural comparison was performed in a study sampling subjects from Denmark, Switzerland, and Poland (Kuettel et al., 2020) to understand the role of the sociocultural context in elite sports athletes' transition. The results showed similarities and differences between countries (Kuettel et al., 2020). Similarly, it was shown that running movement patterns vary between different running groups based on the cultural relevance attributed to running (Wallace et al., 2022).

Despite culture acting as an independent variable in comparative research, in cross-cultural studies the culture is beyond the control of the researchers (van de Vijver and Leung, 2021). When cross-cultural differences are not explained by cultural differences, contextual variables (e.g., economic, social, and demographic factors) are used as a proxy for cultural characteristics (van de Vijver and Leung, 2021). These characteristics present important practical applications for study's design, as will be presented later. For the present purpose, the cross-cultural psychology approach will be used as a framework since our assumption considers that between countries' differences in social, economic, demographic, and cultural characteristics can be related to different behaviors, more precisely the performance in the running context (Segall et al., 1999).

## 4. Foundations for the runners' performance holistic approach

**Figure 1:**
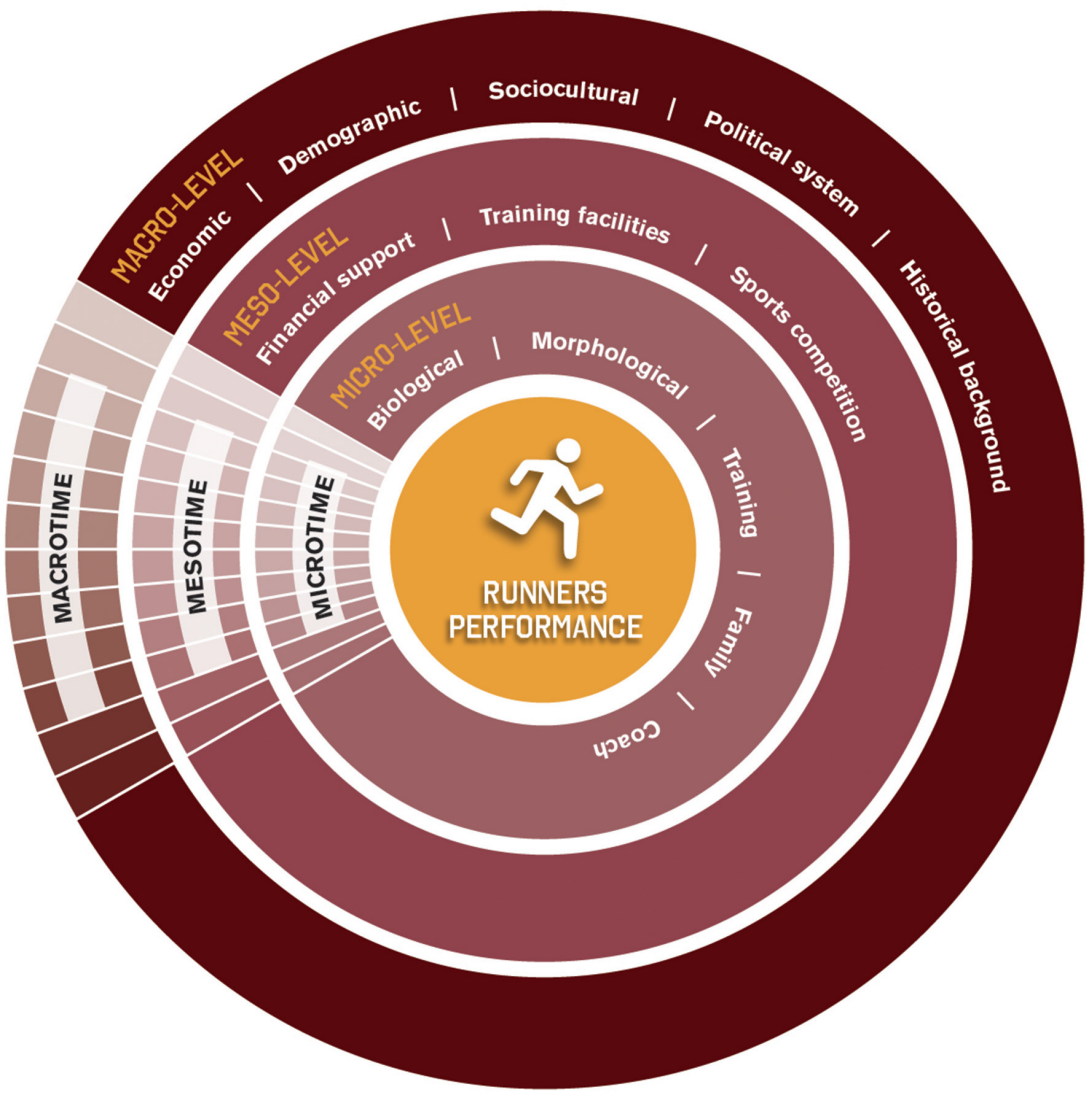
Conceptual model of runners' performance holistic approach [the figure was based on a previous study developed by Thuany et al. (2023)].

Investigating runners' performance through a holistic approach highlights that many factors influence sports success, which is intrinsically related to the athletes and the wider context where they are inserted (Hristovski et al., 2012; Renfree and Casado, 2018). Based on the scientific background that shows the role of the proximal and distal variables in the expression of an athlete's performance, we propose an approach to understanding a runner's performance. This conceptual model was developed to advance the comprehensiveness of this specific topic (Figure 1).

Running performance is the “core” of the investigation, while the cross-sectional line highlights the relationship established between the different levels. The three main aspects of our model are (1) the hierarchical relationship between three different informational levels, (2) the interaction between the different levels, and (3) the relative importance of variables between and within levels for runners' performance. To reduce the complexity and provide some understanding, performance was considered as a product (i.e., running pace, finish time, and ranking position) that emerges from the micro-level, meso-level, and macro-level interactions. The structure indicates a hierarchical organization, where the first level (micro-level) presents variables most directly related to the performance, while the third level (macro-level) brings the variables that are not usually highlighted as closely related to the expression of sports performance, but the role and relevance should be considered both directly and indirectly in athletes' day life.

Furthermore, sports performance is also related to continuity and change throughout athletes' life. For that, the main key in the model is *time* as a predictor. The time as a predictor refers to these changes, which are expected to occur in each variable during a frame of time. Since subject and environment change over time, as a result of different intrapersonal and external experiences, the performance outcomes and the process are continuously changing. As variables situated at different levels act at different timescales (Balagué et al., 2019), microtime, mesotime, and macrotime were presented similarly to the purpose of ecological systems theory. This means that microtime includes changes occurring at a proximal level of the subject, while mesotime refers to the frequency of these changes, and macrotime focuses on the changes located on a large scale of society. On the contrary, some levels of structural stability and depth are expectable within a group (Schein, 2004).

### 4.1. Micro-level

Micro-level comprises variables related to individual characteristics (biology, morphology, and training) and personal environment (athlete–coach relationship and athlete's family). From this lower information level of the model, the scientific literature presents a set of variables related to runners' performance (Alvero-Cruz et al., 2020; Pereira et al., 2021). For the present model, biological (age, sex), morphological [body mass index (BMI)/body composition], and training variables (frequency, volume, time of practice, and training methods used) were considered, based on previous studies that show their direct or indirect role to the expression of runners' performance (Casado et al., 2019; Alvero-Cruz et al., 2020).

Among these variables, anthropometric and body composition are the most investigated, possibly due to the low cost and practicality associated with their measurement. In general, the results show that there is a negative relationship between BMI and fat percentage with running performance (Sedeaud et al., 2014; Vincent et al., 2020; Thuany et al., 2022). Moreover, these results may be explained by factors related to the following characteristics: (a) running is considered a “weight-sensitive” sports practice (Sedeaud et al., 2014; Vincent et al., 2020), where the generation of force to sustain the body weight during displacement is the primary determinant of the metabolic cost of running, and a “simple” increase of 10% in body mass may represent an increase of ~14% in running energy expenditure (Silva, 2019); and (b) the fact that ~20% of the energy spent during displacement is for the acceleration of the lower limbs, so an increase in body mass may lead to loss of efficiency, as well as greater heat accumulation at a given submaximal running speed, which may compromise the exercise if high internal temperatures are reached (Fuziki, 2012).

In addition, we should not ignore the intersection between anthropometric variables, body composition, and training characteristics (Thuany et al., 2022). The running speed during training sections and the body fat percentage explains ~44% of the performance of recreational marathon runners (Gómez-Molina et al., 2017). In contrast, the association of practice time, training volume, BMI, and skinfold sum explains ~90.3% of the variance in the half-marathon performance among non-professional male runners (Barandun et al., 2012). Therefore, biological, morphological, and training variables constantly interact to the expression of running performance (Thuany et al., 2022).

The role of the family and coach in athletes' performance was previously mentioned. Parents and coaches are the first socialization agents for sports participation and performance (Luo and Kiewra, 2020; Tessitore et al., 2021). They are responsible to transfer standards and values, providing encouragement, moral and social support, and acting as role models for sports participation. In addition to that, financial support for the acquisition of training equipment, participation in competitions in different places, and nutritional consumption are also important aspects that cannot be neglected. Similarly, coaches also encourage when athletes faced stiff competition, providing advice on the best way to succeed in sports through hard work and perseverance, providing psychological support through motivation, and spiritual support through prayers toward success and achievement (Lassalle et al., 2018). Furthermore, a study conducted with Kenyan runners' families showed that family played an important role in the development of athletic talent and influencing their performance (Mwanga et al., 2017). The same relationship was observed for training facilities and participation; however, more studies are necessary to understand how family members, coaches, and teammates influence training commitment, athletes' development, and performance.

### 4.2. Meso-level

In the Bronfenbrenner (1977, 2011) ecological systems theory, the meso-level is related to the environment in which subjects are not directly exposed but are directly influenced. For the present proposal, meso-level variables are less explored in the context of running performance, and for that, the variables included in the present model are related to the training environment, considering both financial support and facilities and sports competition. For young athletes' development process, the role played by financial support for training, competition participation, and full-time dedication was highlighted.

For runners, information about financial support and training facilities that are associated with running performance is limited. Endurance running is traditionally known as a discipline where sophisticated equipment is not mandatory, and the main training is performed in outdoor spaces, not requiring access and sponsorship. Despite these characteristics, resistance training—previously associated with performance outcomes in runners (Blagrove et al., 2018)—is performed in gymnasiums, sports arenas, or training centers, limiting access for some runners. In addition, participation in competitions (part of the athletes' routine) involves human, material, and financial resources. Given that some evidence shows that social ascension was related to African runners' motivation and search for performance (Onywera et al., 2006; Elbe et al., 2010), advances about the role of competition in motivation and training maintenance, as well as the role of financial support and training facilities to push the environment, in which the athlete is committed should be considered in future studies. In addition, it is necessary to fill the gaps regarding variables that can connect micro-level and macro-level.

### 4.3. Macro-level

Macro-level factors consider environmental features that shape the sports systems. For the present model, the macro-level comprises the economic, social, cultural, and demographic domains. In terms of influence on running performance, a direct relationship between the macro level and athletes' performance is not expectable, instead, the influence on the expression of variables situated at the meso-level and macro-level should be considered. Most of the evidence about the macro-level variables is based on studies comparing countries in the international sports context, such as Olympic Games and World Championships (Truyens et al., 2014; De Bosscher et al., 2015). Demographic factors (population size and human development index), political system, and income explained more than half of the countries' success (Bohme and Bastos, 2016). The results suggested that outputs (i.e., an Olympic medal) are different due to different inputs (e.g., economic characteristics).

Beyond these variables, shared culture and beliefs within a country can also be related to the commitment to different sports practices and performances (Bale and Sang, 1996). Rothwell et al. (2018) refer to behaviors, attitudes, beliefs, and values that shape the communities with the potential to influence the development of sports performance. However, the magnitude in which the cultural context can reduce the role of economic, demographic, and political variables is unknown, and limited information is available for specific sports disciplines (Gomes-Sentone et al., 2019; Santos et al., 2019). For example, Mazzei et al. (2023) showed that for judo, the results in previous events were the most important variables to explain the countries' performance competing in the Olympic games between 1992 and 2016. These results were contextualized considering historical and socioeconomic differences. Investigating the frequency of countries in the World Athletics ranking for sprint and endurance race events, Santos et al. (2019) showed that most of the countries ranked among endurance events are classified as medium or lower income. This result is associated with the African phenomenon. For example, Kenya and Ethiopia are ranked at 141° and 171° positions, respectively, on the human development index board, but they produce the highest number of elite endurance runners worldwide.

In addition to these aspects, other factors are documented, such as political, religious, and cultural systems and social, demographic, and sports organizations. The conclusions of these studies suggest that there is little information about the role of macro-level variables on the programs for participation in sports, and they highlight the importance of this information for decision-making on the managerial and pedagogical levels. In this sense, macro-level variables, such as environmental characteristics, politics, geographical disposition, and shared beliefs, can be related to the likelihood of participating in sports practice, training opportunities, and performance development.

## 5. Discussion

At the heart of our conceptual model, runners' performance is an outcome of an imbricated relationship between variables situated at different levels. Beyond individual factors related to runners' performance, future studies need to investigate running performance as a result of the relationship between subject and environment. For that, the use of different research strategies should be considered in order to provide some understanding of the pattern of interactions between different variables in specific contexts. Models based on geographic or social boundaries should be considered using cross-cultural research as a framework to guide practice. In association, changes occurring during athletes' life, as a result of personal experience, as well as those resulting from external forces must be considered. Since predicting human behavior is a challenging task, this model is not static, and neither is our purpose to provide a deterministic approach.

A better understanding of the role of environmental factors in runners' performance and being able to identify variables that connect different levels can be useful at different theoretical and practical levels. In this sense, practical applications of the present conceptual model include the creation of a nurturing environment, considering the individual characteristics in association with the environmental features, to provide good development (i.e., performance and training commitment) for all participants. In addition, the consideration of a specific environment can be useful to create a more equal and empowering environment for everyone if we acknowledge the influence of culture and social context on runners' experiences and outcomes.

For this task, a deep comprehension of the role of the time and the culture should be considered. Even though culture is also considered a complex group learning process, culture should be contextualized and investigated at different levels, including micro-level and meso-level (Schein, 2004). As one of the main social phenomena around the world, the potential of sport to act as a catalysator for human development should not be neglected. For some countries and athletes, sports are related to the possibility of economic and social ascension. These characteristics may reinforce the idea that “taking some risks” is part of the process of becoming an athlete; however, in addition to the athlete's development process, human development needs to be considered, indicating that better conditions to be engaged in training and competition are important for both outcomes: sports performance and life.

Despite the ecological systems theory being previously used as a framework in different sports contexts, some advances are mandatory. Most of these advances are related to synergic, multidisciplinary, and collaborative work between academics, stakeholders, and athletes of different places and working in different settings. Researchers with an interest to understand running context through an ecological framework are invited to use our suggestion as a starting line to understand how the subject and environment interact with each other to better express performance outcomes. As an unfinished study, we hope to be able to advance our current knowledge, advancing and filling some gaps related to the role of different environmental settings in different performance outcomes, as well as understand countries' specificities which can be of relevance for the development of friendlier environments for sports and human development.

## Data availability statement

The original contributions presented in the study are included in the article/supplementary material, further inquiries can be directed to the corresponding author.

## Author contributions

MT worked on the original draft preparation and review/editing. TG, KW, BK, RR, and MM revised the text. RR, TG, and MM supervised and worked on the review and editing of the manuscript.
